# Relationship between depression improvement and activities of daily living recovery in patients with fractures

**DOI:** 10.1002/pcn5.70139

**Published:** 2025-06-24

**Authors:** Shunji Araki, Takahiro Ogawa, Yoshikazu Takaesu

**Affiliations:** ^1^ Chuzan Hospital Clinical Education and Research Center Okinawa Japan; ^2^ Department of Rehabilitation Medicine Aichi Medical University Nagakute Japan; ^3^ Department of Neuropsychiatry, Graduate School of Medicine University of the Ryukyus Okinawa Japan

**Keywords:** activities of daily living (ADL), depression, Geriatric Depression Scale (GDS), older patients with fractures, rehabilitation

## Abstract

**Aim:**

This study aimed to investigate the relationship between improvement in depressive symptoms and activities of daily living (ADL) recovery in older patients with fractures in convalescent rehabilitation wards.

**Methods:**

A retrospective observational study was conducted with 144 older patients with fractures and depressive symptoms (Geriatric Depression Scale score ≥ 5) on admission, who underwent rehabilitation at a Japanese hospital from 2018 to 2023. ADL recovery was assessed by the Functional Independence Measure (FIM). Patients were classified into three groups based on change in depressive symptoms: remission, partial improvement, and no improvement. Multivariate linear regression analysis was used to evaluate factors associated with change in the FIM score.

**Results:**

Remission (*β* = 0.184, *p* = 0.019) and partial improvement in depressive symptoms (*β* = 0.204, *p* = 0.009) were positively associated with change in the FIM score. Conversely, antidepressant use (*β* = −0.250, *p* = 0.002) was negatively associated with change in the FIM score.

**Conclusion:**

Remission and partial improvement in depressive symptoms positively contributes to ADL recovery in older patients with fractures. Comprehensive interventions addressing both depression and physical function may enhance rehabilitation outcomes. This may improve ADL recovery and long‐term prognosis in older patients with fractures.

## INTRODUCTION

As individuals age, bone fragility increases, leading to a heightened risk of fractures. In adults over the age of 50, 53% of fractures are attributed to low‐impact trauma resulting from bone fragility.^1^ The most common sites of fragility fractures are the hip, vertebrae, and distal radius.^2^ Recently, there have been increasing reports of pelvic ring fractures associated with aging.^3^, ^4^, ^5^ Regarding hip fractures, an analysis conducted across 19 countries and regions from 2005 to 2018 revealed age‐ and sex‐standardized incidence rates ranging from 95.1 to 315.9 per 100,000 population aged 50 years and older. Although the incidence rates of hip fractures have declined in most countries in recent years, the absolute number of fractures continues to rise due to global population aging; it is projected to double by 2050.^6^ Hip fractures significantly impair activities of daily living (ADL), which are strong predictors of mortality.^7^ Excess mortality associated with hip fractures ranges from 8.4% to 36% in the first year after the fracture.^1^ Similarly, vertebral fractures, another type of fragility fracture, can lead to morbidity rates comparable to or exceeding those of hip fractures.^8^, ^9^ The prevalence of vertebral fractures in older adults ranges from 18% to 51%, particularly among women, and increases with age. Among adults aged 65 years or older, vertebral fractures are associated with a significantly reduced long‐term survival rate, with mortality risk six times higher than that of the general population.^10^ Rehabilitation, including exercise, plays a crucial role in improving physical function, ADL, and postoperative complications in patients with fractures.^11^, ^12^, ^13^ Therefore, rehabilitation is essential for improving the long‐term prognosis of patients with fractures.

Late‐life depression is a common mental health disorder that negatively affects health‐related quality of life. Major depression occurs in 2% of adults aged 55 years or older, with its prevalence increasing with age. Additionally, 10% to 15% of older adults experience depressive symptoms without meeting the criteria for major depression.^14^ Depression is the most prevalent mental health condition in older adults, yet it is often underdiagnosed and undertreated.^15^ Studies involving community‐dwelling older adults have demonstrated that depressive symptoms are associated with subsequent decline in physical functioning.^16^, ^17^, ^18^

The association between fractures and depression has been reported, with 9% to 47% of patients with hip fractures having depression,^19^ and most patients with fragility fractures having depression.^20^ Comorbid depressive symptoms in patients with hip and other fragility fractures reduce subsequent improvement in ADL and physical function and are associated with excess mortality.^20^, ^21^, ^22^ In other words, depressive symptoms comorbid in patients with fractures decrease rehabilitation effectiveness,^23^ and it is inferred that improving depressive symptoms may increase rehabilitation effectiveness. Although studies have investigated the effect of comorbid depressive symptoms on admission and rehabilitation effectiveness, mainly in patients with hip fractures, no research has focused on the improvement in depressive symptoms and rehabilitation effectiveness in patients with fractures admitted to convalescent rehabilitation wards. One of the outcomes of rehabilitation for patients with fractures is ADL recovery,^24^, ^25^ and ADL recovery has been associated with subsequent physical function and mortality.^26^, ^27^, ^28^, ^29^, ^30^ Therefore, ADL recovery is an important rehabilitation outcome for patients with fractures. Identifying the association between the improvement in depressive symptoms and ADL recovery may provide an opportunity to focus on depressive symptoms in the rehabilitation of patients with fractures. Addressing depressive symptoms in addition to rehabilitation for physical function and ADL may enhance the effectiveness of rehabilitation and improve the long‐term prognosis of patients with fractures. Therefore, the purpose of this study was to examine the relationship between the improvement in depressive symptoms and ADL recovery in older patients with fractures and depression admitted to convalescent rehabilitation wards.

## METHODS

### Study design and participants

This retrospective, observational study used data routinely collected at a rehabilitation hospital in Okinawa, Japan from August 2018 to January 2023.

The participants were patients admitted for rehabilitation after fracture treatment, with depressive symptoms on admission. The inclusion criteria were as follows: (1) admission and discharge for rehabilitation after fracture treatment from August 2018 to January 2023; and (2) Geriatric Depression Scale (GDS) score ≥ 5 on admission. The exclusion criteria were as follows: (1) inability to take bioelectrical impedance analysis (BIA) to accurately measure skeletal muscle mass, such as patients who had cardiac pacemakers or who could not stay sufficiently calm during BIA; (2) discharge due to the onset of acute diseases; (3) death during hospitalization; (4) missing data on GDS, energy intake, and Mini Nutritional Assessment Short Form (MNA‐SF) results; and (5) a Mini‐Mental State Examination (MMSE) score <10, as GDS is less reliable in patients with cognitive decline.^31^

### Rehabilitation program

In these wards, patients with fractures receive approximately 60–180 min of rehabilitation per day for 1–3 months. All the participants received rehabilitation as instructed by medical doctors and were required to undergo rehabilitation with therapists every day, including training in muscle strength and ADL. Additionally, they underwent sitting and standing training, balance training, gait exercises, stepping exercises, and training to improve their ability to live their daily lives. Based on their individual needs, participants used corsets, orthoses, and canes while exercising or whenever necessary. Some participants underwent rehabilitation with only partial or no body weight load on the fractured part. This depended on their condition. The participants were advised not to perform rehabilitation activities when their circulation or breathing was impaired, or if their condition worsened.

### Ethical considerations

The study was approved by the Ethics Review Committee for Life Sciences and Medical Research Involving Human Subjects, University of the Ryukyus (approval ID: 23‐2256‐00‐00‐00). Regardless of whether they could be included in this study, all patients were required to undergo GDS assessment in this hospital to evaluate the effectiveness of the rehabilitation programs. Since this study was retrospective in design, an opt‐out procedure was used to provide all participants with the opportunity to exclude their data from the study analysis. This study was conducted in accordance with the principles of the Declaration of Helsinki.

### Data collection

Clinical data on admission and at discharge were retrospectively collected from the clinical database. This included data on age, sex, fracture type, body mass index (BMI), MNA‐SF, Charlson Comorbidity Index (CCI),^32^, ^33^, ^34^ MMSE, energy intake, protein intake, skeletal muscle mass index (SMI), GDS, Functional Independence Measure (FIM), length of hospital stay for rehabilitation, daily rehabilitation period, and the use of antidepressants.

The BMI was calculated as the weight in kilograms divided by the height in meters squared. Nutritional status was assessed using the MNA‐SF, with scores ranging from 1 to 14. Normal nutritional status, risk of malnutrition, and malnutrition were indicated by scores of ≥12, 8–11, and <8, respectively.^35^

The CCI remains the most widely validated and used comorbidity score and includes 17 conditions: myocardial infarction, congestive heart failure, peripheral vascular disease, cerebrovascular disease, dementia, chronic pulmonary disease, connective tissue disease, peptic ulcer disease, mild liver disease, and diabetes each scored 1; hemiplegia, moderate or severe renal disease, diabetes with end organ damage, any tumor, leukemia, and lymphoma each scored 2; moderate or severe liver disease each scored 3; and metastatic solid tumors or acquired immune deficiency syndrome each scored 6.

Cognitive function was assessed using the MMSE,^36^ a widely used tool containing 11 items that assess orientation, registration, attention and calculation, recall, and language.^37^ MMSE scores range from 0 to 30, with lower scores indicating more severe cognitive impairment.

Energy and protein intake were obtained from clinical records, and included oral, intravenous, and enteral nutrition. Nurses or registered dieticians evaluated the remaining energy and protein intake after each meal, and calculated the energy and protein intakes accordingly.

All patients underwent BIA using a segmental multifrequency bioelectrical impedance analyzer (InBody S10; InBody Japan). Multifrequency impedance body composition analysis has been shown to correlate well with the results of dual‐energy X‐ray absorptiometry, and has been validated.^38^, ^39^, ^40^, ^41^ Patients rested quietly in a neutral supine position ≥15 min prior to BIA measurements. Skeletal muscle mass was estimated using BIA measurements, and the SMI was then calculated as the skeletal muscle mass divided by the square of the height.^42^

### Symptoms of depression

Depressive symptoms were assessed using the 15‐item GDS.^43^, ^44^ This scale was translated into Japanese and a validation study was conducted with Japanese participants. As a result, the Japanese version of the GDS proved to be a clinically applicable screening instrument for depression, regardless of age or sex.^45^, ^46^ The GDS scale ranges from 0 to 15 points and expresses the severity of depressive symptoms. GDS scores ≤ 4 denote the absence of depressive symptoms and GDS scores ≥ 5 indicate the presence of depressive symptoms.^47^ The GDS was assessed on admission and discharge by trained therapists. Change in depressive symptoms was determined by subtracting the GDS score on admission from the GDS score at discharge. Hence, a zero‐change score denotes no change in depressive symptoms, a negative change score denotes an improved symptom level, and a positive change score denotes worsening depressive symptoms. In this study, we defined the remission group as those with GDS scores ≤ 4 at discharge, the partial‐improvement group as those with GDS scores at discharge lower than those on admission but ≥ 5 at discharge, and the no‐improvement group as those with GDS scores at discharge equal to or higher than those on admission, based on previous studies.^47^

### Ability to perform ADL

ADL was assessed using the FIM.^48^ This scale ranges from 18–126 points, and is based on a score of 1 to 7 for each of 18 items in accordance with the level of independence in performing the item. In this scale, total autonomy is reflected by a score of 126 points. The scale is divided into six subscales: Self‐Care, Sphincter Control, Transfers, Locomotion, Communication, and Social Cognition. Self‐Care, Sphincter Control, Transfers, and Locomotion are the motor FIM domain. Each item was scored from 1 (*total dependence*) to 7 (*complete independence*). The FIM was assessed on admission and discharge by a nurse. Change in the FIM score was calculated by subtracting the FIM on admission from the FIM at discharge. Therefore, with respect to change in the FIM score, conversely, a zero or negative change score indicates no change or worse ADL, and a positive change score indicates improved ADL.

### Statistical analysis

All statistical analyses were performed using EZR (Saitama Medical Center, Jichi Medical University), a graphical user interface for R (The R Foundation for Statistical Computing). Specifically, this is a modified version of R commander designed to add statistical functions frequently used in biostatistics. All continuous variables are presented as medians with interquartile ranges, and were compared across the three groups using the Kruskal–Wallis test. For the post‐hoc tests, we used the Steel–Dwass test. All categorical variables are expressed as the number of patients and percentages, and were compared across the three groups using Fisher's exact test.

First, we compared the change in FIM score among the three groups based on their depressive symptoms and status at discharge. Differences among the three groups were analyzed using the Steel–Dwass test. Additionally, to clarify the association between change in the FIM score and the three groups, we performed multivariate linear regression analysis with change in the FIM score as the objective variable. The explanatory variables were entered incrementally into Models 1, 2, and 3. Model 1 represents univariate linear regression analyses conducted separately for remission and partial improvement in depressive symptoms to assess their crude associations with change in FIM score. Model 2 included the MMSE and CCI as additional explanatory variables, based on previous studies suggesting their association with ADL outcomes. Finally, Model 3 included additional variables that had *p* < 0.1 in the univariate linear regression analyses and were considered potential predictors of change in FIM score.^49^ All statistically significant differences were evaluated at *p* < 0.05.

## RESULTS

**Figure 1:**
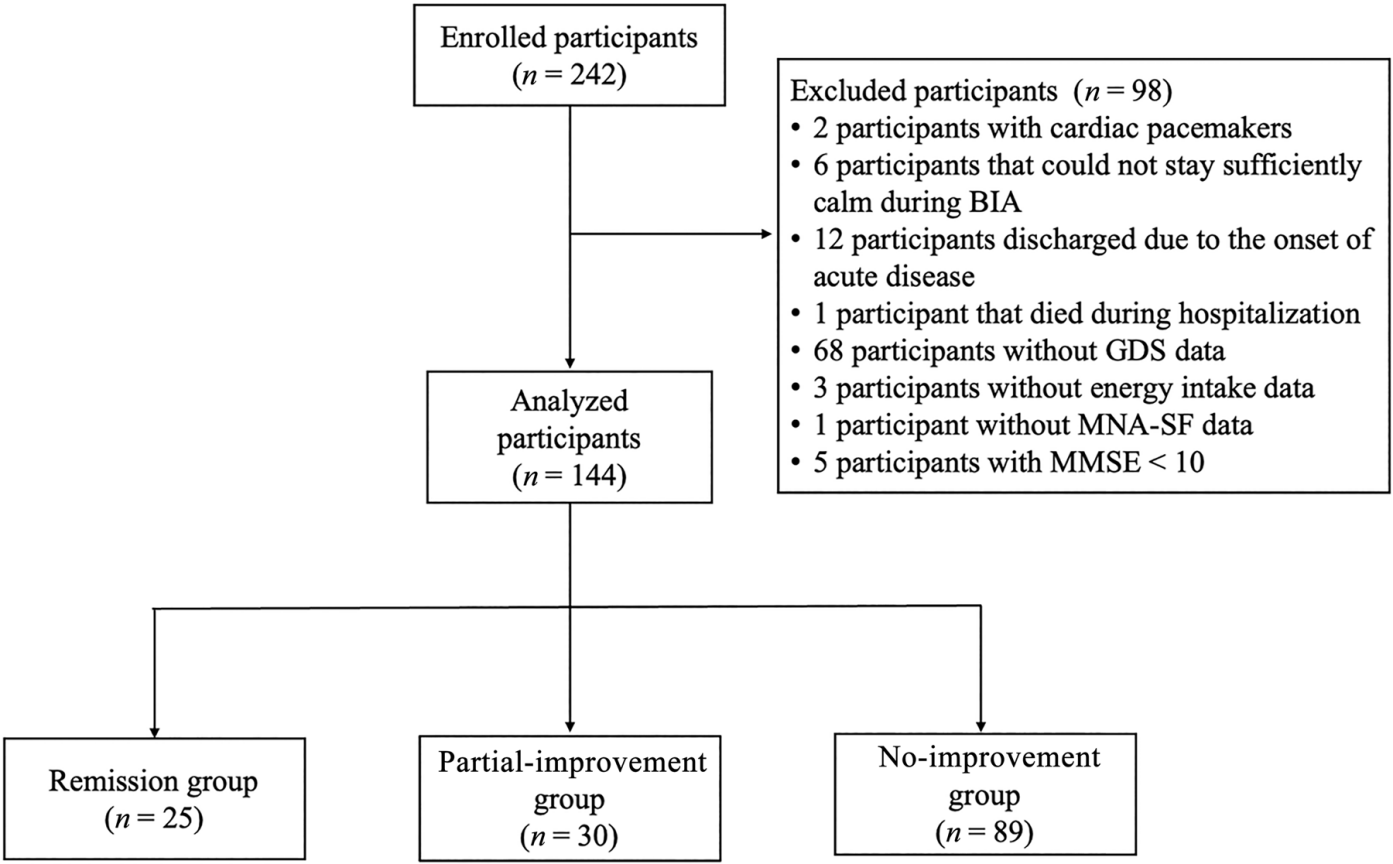
Flowchart of the study population. The remission group included participants with a Geriatric Depression Scale (GDS) score ≤4 at discharge. The partial‐improvement group included participants with a GDS score at discharge lower than that on admission, but ≥5 at discharge. The no‐improvement group included participants with a GDS score at discharge equal to or higher than that on admission. BIA, bioelectrical impedance analysis; MMSE, Mini‐Mental State Examination; MNA‐SF, Mini Nutritional Assessment Short Form.

A total of 242 consecutive patients with fractures were initially eligible for enrollment in this study; however, 98 met the exclusion criteria (Figure 1). Of these, two had cardiac pacemakers; four on admission and two at discharge could not stay sufficiently calm during BIA; 12 were discharged due to onset of acute disease; one died during hospitalization; 72 had missing data on GDS, energy intake, and MNA‐SF; and five had MMSE scores <10. Therefore, the final analysis included 144 patients.

Table 1 shows the baseline characteristics of the study participants. The median age, GDS score, and FIM score of the participants were 83.0 (range: 77.8–87.0) years, 8.0 (range: 6.0–10.0), and 63.5 (range: 54.0–73.0), respectively.

**Table 1:** Study sample characteristics at baseline.

	Whole sample (*N* = 144)
Age, years	83.0 (77.8–87.0)
Sex, *n* (%)	
Male	35 (24.3)
Female	109 (75.7)
Fracture types, *n* (%)	
Femoral fracture	78 (54.2)
Vertebral fracture	49 (34.0)
Others	17 (11.8)
BMI, kg/m^2^	22.8 (20.1–24.5)
FIM	63.5 (54.0–73.0)
Motor FIM	40.0 (32.0–47.0)
MMSE	20.0 (16.0–24.0)
GDS	8.0 (6.0–10.0)
SMI, kg/m^2^	5.3 (4.6–6.0)
CCI	2.0 (1.0–3.0)
MNA‐SF	8.0 (6.0–10.0)
Antidepressant use, *n* (%)	
Use	23 (16.0)
No use	121 (84.0)

*Note*: Values are presented as medians with interquartile ranges.

Abbreviations: BMI, body mass index; CCI, charlson comorbidity index; FIM, functional independence measure; GDS, geriatric depression scale; MMSE, mini‐mental state examination; MNA‐SF, mini nutritional assessment short form; SMI, skeletal muscle mass index.

Table 2 shows a comparison of variables on admission of the three groups. The median GDS score was 6.0 (range: 5.0–10.0) for the remission group, 10.0 (range: 9.0–12.0) for the partial‐improvement group, and 8.0 (range: 6.0–9.0) for the no‐improvement group. The median FIM score was 68.0 (range: 61.0–76.0) for the remission group, 59.0 (range: 51.3–65.8) for the partial‐improvement group, and 64.0 (range: 54.0–73.0) for the no‐improvement group. The Kruskal–Wallis test showed significant differences in the FIM score (*p* = 0.035), motor FIM score (*p* = 0.022), and GDS score (*p* < 0.001) among the three groups. In the post‐hoc test, the FIM score (*p* < 0.05) showed a significant difference between the remission and partial‐improvement groups, while the motor FIM (*p* < 0.05) and GDS scores (*p* < 0.05) showed a significant difference between the remission and partial‐improvement groups, and between the partial improvement and no‐improvement groups.

**Table 2:** Comparison of variables on admission in three groups.

	Remission group^a^ (*n* = 25)	Partial‐improvement group^b^ (*n* = 30)	No improvement group^c^ (*n* = 89)	*p* value
Age, years	84.0 (81.0–88.0)	82.5 (77.3–87.0)	83.0 (76.0–87.0)	0.448
Sex, *n* (%)				0.271
Male	3 (12.0)	7 (23.3)	25 (28.1)	
Female	22 (88.0)	23 (76.7)	64 (71.9)	
Fracture types, *n* (%)				
Femoral fracture	11 (44.0)	15 (50.0)	52 (58.4)	0.390
Vertebral fracture	10 (40.0)	11 (36.7)	28 (31.5)	0.700
Others	4 (16.0)	4 (13.3)	9 (10.1)	0.662
BMI, kg/m^2^	24.1 (20.3–25.5)	22.7 (21.1–24.4)	22.5 (20.0–24.3)	0.479
FIM	68.0 (61.0–76.0)	59.0 (51.3–65.8)	64.0 (54.0–73.0)	0.035*
Motor FIM	44.0 (38.0–48.0)	35.5 (29.0–43.0)	40.0 (34.0–47.0)	0.022* ^,^ †
MMSE	24.0 (19.0–26.0)	19.5 (15.3–23.8)	19.0 (16.0–23.0)	0.091
GDS	6.0 (5.0–10.0)	10.0 (9.0–12.0)	8.0 (6.0–9.0)	<0.001* ^,^ †
SMI, kg/m^2^	5.3 (5.1–5.9)	4.9 (4.1–5.6)	5.4 (4.6–6.0)	0.191
CCI	1.0 (1.0–2.0)	2.0 (1.0–2.0)	2.0 (1.0–3.0)	0.459
MNA‐SF	9.0 (7.0–10.0)	7.0 (6.0–9.0)	8.0 (6.0–10.0)	0.236
Antidepressant use, *n* (%)				
Use	3 (12.0)	4 (13.3)	16 (18.0)	0.851
No use	22 (88.0)	26 (86.7)	73 (82.0)	

*Note*: Values are presented as medians with interquartile ranges.

Abbreviations: BMI, body mass index; CCI, charlson comorbidity index; FIM, functional independence measure; GDS, geriatric depression scale; MMSE, mini‐mental state examination; MNA‐SF, mini nutritional assessment short form; SMI, skeletal muscle mass index.

^a^ The remission group included participants with a GDS score ≤4 at discharge.

^b^ The partial‐improvement group included participants with a GDS score at discharge lower than that on admission but ≥5 at discharge.

^c^ The no‐improvement group included participants with a GDS score at discharge equal to or higher than that on admission.

Post hoc analysis:

* *p* < 0.05 (remission group versus partial‐improvement group).

^†^ *p* < 0.05 (partial‐improvement group versus no‐improvement group).

Table 3 shows a comparison of variables at discharge of the three groups. The median change in the GDS score was −5.0 (range: −7.0–−2.0) for the remission group, −3.0 (range: −4.0–−2.0) for the partial‐improvement group, and 0.0 (range: 0.0–0.0) for the no‐improvement group. The median change in the FIM score was 44.0 (range: 37.0–49.0) for the remission group, 40.0 (range: 26.5–55.0) for the partial‐improvement group, and 33.0 (range: 21.0–43.0) for the no‐improvement group. The Kruskal–Wallis test showed significant differences in FIM (*p* = 0.006), change in FIM (*p* = 0.001), motor FIM (*p* = 0.010), change in motor FIM (*p* < 0.001), GDS scores (*p* < 0.001), and change in GDS scores (*p* < 0.001). In addition, the Steel–Dwass post‐hoc test indicated that the remission group had a significantly greater change in FIM score than did the no‐improvement group (*p* < 0.05).

**Table 3:** Comparison of variables at discharge in three groups.

	Remission group^a^ (*n* = 25)	Partial‐improvement group^b^ (*n* = 30)	No improvement group^c^ (*n* = 89)	*p* value
Period of rehabilitation, min/day	128.6 (116.2–146.6)	122.1 (109.7–137.8)	125.0 (114.0–140.4)	0.656
Length of hospital stay, days	71.0 (43.0–83.0)	71.0 (56.5–83.5)	67.0 (50.0–84.0)	0.876
FIM	113.0 (104.0–119.0)	105.0 (81.5–120.0)	101.0 (80.0–112.0)	0.006‡
Change in FIM score^d^	44.0 (37.0–49.0)	40.0 (26.5–55.0)	33.0 (21.0–43.0)	0.001‡
Motor FIM	84.0 (77.0–87.0)	72.5 (60.0–87.8)	73.0 (58.0–84.0)	0.010‡
Change in motor FIM score^e^	38.0 (34.0–44.0)	36.0 (25.3–44.8)	29.0 (21.0–36.0)	<0.001‡
GDS	3.0 (2.0–3.0)	7.0 (5.3–8.0)	8.0 (6.0–11.0)	<0.001* ^,^ † ^,^ ‡
Change in GDS score^f^	−5.0 (−7.0–−2.0)	−3.0 (−4.0–−2.0)	0.0 (0.0–0.0)	<0.001* ^,^ ‡
SMI, kg/m^2^	5.6 (5.2–6.1)	4.9 (4.2–5.9)	5.3 (4.7–6.2)	0.134
Change in SMI,^g^ kg/m^2^	0.3 (0.1–0.5)	0.2 (0.0–0.4)	0.1 (0.1–0.3)	0.066
Energy intake, kcal	1440.0 (1274.0–1600.0)	1400.0 (1363.0–1600.0)	1440.0 (1280.0–1600.0)	0.891
Protein intake, g/kg/day	1.2 (1.0–1.3)	1.2 (1.1–1.3)	1.1 (1.0–1.3)	0.681

*Note*: Values are presented as medians with interquartile ranges.

Abbreviations: FIM, functional independence measure; GDS, geriatric depression scale; SMI, skeletal muscle mass index.

^a^ The remission group included participants with a GDS score ≤ 4 at discharge.

^b^ The partial‐improvement group included participants with a GDS score at discharge lower than that on admission but ≥ 5 at discharge.

^c^ The no‐improvement group included participants with a GDS score at discharge equal to or higher than that on admission.

^d^ The change in the FIM score was obtained by subtracting the FIM score on admission from the FIM score at discharge.

^e^ The change in the motor FIM score was obtained by subtracting the motor FIM score on admission from the motor FIM score at discharge.

^f^ The change in the GDS score was obtained by subtracting the GDS score on admission from GDS score at discharge.

^g^ The change in the SMI was obtained by subtracting the SMI on admission from the SMI at discharge.

Post hoc analysis:

* *p* < 0.05 (remission group versus partial‐improvement group).

^†^ *p* < 0.05 (partial‐improvement group versus no‐improvement group).

^‡^ *p* < 0.05 (remission group versus no‐improvement group).

Table 4 shows the associations between change in the FIM score with remission and partial improvement in depressive symptoms in multivariate linear regression analysis with other potential predictors. In Model 1, remission (*β* = 0.210, *p* = 0.011) and partial improvement in depressive symptoms (*β* = 0.166, *p* = 0.047) were each significantly associated with change in FIM score, as shown in separate univariate linear regression analyses. In Model 2, remission (*β* = 0.220, *p* = 0.008), partial improvement in depressive symptoms (*β* = 0.228, *p* = 0.005), and MMSE on admission (*β* = 0.214, *p* = 0.009) were significantly associated with change in FIM score. In Model 3, BMI on admission, fracture type (femoral fracture, vertebral fracture), use of antidepressants, period of rehabilitation, and SMI on admission were selected as potential predictors in univariate linear regression analyses. Remission (*β* = 0.184, *p* = 0.019), partial improvement in depressive symptoms (*β* = 0.204, *p* = 0.009), and the use of antidepressants (*β* = −0.250, *p* = 0.002) were significantly associated with change in FIM score. Both remission and partial improvement in depressive symptoms were positively associated with change in FIM score, while the use of antidepressants was negatively associated with change in FIM score.

**Table 4:** Univariate and multivariate linear regression analysis for change in the FIM score.^a^

Factor	Model 1 (univariate)		Model 2 (multivariate)		Model 3 (multivariate)	
	*β* (95% CI)	*p* value	*β* (95% CI)	*p* value	*β* (95% CI)	*p* value
Remission in depressive symptoms^b^	0.210 (0.048 to 0.372)	0.011	0.220 (0.059 to 0.381)	0.008	0.184 (0.031 to 0.336)	0.019
Partial improvement in depressive symptoms^c^	0.166 (0.002 to 0.329)	0.047	0.228 (0.069 to 0.387)	0.005	0.204 (0.052 to 0.356)	0.009
No improvement in depressive symptoms^d^ (reference)	−	−	−	−	−	−
MMSE on admission	−	−	0.214 (0.053 to 0.75)	0.009	0.131 (−0.030 to 0.292)	0.110
CCI on admission	−	−	−0.093 (−0.252 to 0.066)	0.250	−0.105 (−0.259 to 0.049)	0.178
BMI on admission, kg/m^2^	−	−	−	−	0.035 (−0.159 to 0.230)	0.721
Femoral fracture^e^	−	−	−	−	−0.220 (−0.455 to 0.015)	0.066
Vertebral fracture^f^	−	−	−	−	−0.080 (−0.316 to 0.155)	0.501
Use of antidepressants^g^	−	−	−	−	−0.250 (−0.405 to −0.095)	0.002
Period of rehabilitation, min/day	−	−	−	−	0.146 (−0.007 to 0.299)	0.061
SMI on admission, kg/m^2^	−	−	−	−	0.031 (−0.167 to 0.229)	0.758

*Note*: Adjusted *R*‐squared (*R* ^2^) model: Model 1 = 0.037 (remission), 0.020 (partial improvement); Model 2 = 0.131; Model 3 = 0.239.

Model 1 represents univariate linear regression analyses conducted separately for remission and partial improvement in depressive symptoms, to assess their crude associations with change in FIM scores.

Model 2 included MMSE and CCI as additional explanatory variables.

Model 3 further added variables with *p* < 0.1 in the univariate analysis as potential predictors.

Abbreviations: β, standardized beta; BMI, body mass index; CCI, charlson comorbidity index; CI, confidence interval; FIM, functional independence measure; MMSE, mini‐mental state examination; SMI, skeletal muscle mass index.

^a^ The change in the FIM score was obtained by subtracting the FIM score on admission from the FIM score at discharge.

^b^ Participants with GDS scores ≤4 at discharge are coded as 1; all others are 0.

^c^ Participants with GDS scores at discharge lower than on admission, but ≥5 at discharge are coded as 1; all others are 0.

^d^ Participants with GDS scores at discharge equal to or higher than those on admission.

^e^ Femoral fracture is coded as 1; all others are 0.

^f^ Vertebral fracture is coded as 1; all others are 0.

^g^ Use of antidepressants is coded as 1; no use is 0.

## DISCUSSION

This study is the first to show that, in older patients with fractures and depression, even partial improvement in depressive symptoms significantly contributes to ADL recovery. This is in addition to the improvement from remission. While previous studies have investigated the relationship between depression and ADL recovery,^47^, ^50^ they primarily focused on community‐dwelling older adults without fractures, or patients with fractures irrespective of depressive symptoms. Consequently, the influence of the improvement of depressive symptoms on ADL recovery in older patients with fractures has not been fully explored. This study specifically aimed to address this gap by focusing on patients with fractures and depression in convalescent rehabilitation wards. Changes in FIM score were used as the primary outcome measure. Our results showed that even partial improvement in depressive symptoms was significantly associated with ADL recovery. These findings highlight the critical role of comprehensive rehabilitation programs that address both physical function and depressive symptoms in promoting ADL recovery among older patients with fractures.

The mechanisms underlying the association between partial improvement in depressive symptoms and ADL recovery were explored by comparing our findings with those of previous studies. For instance, Nyunt et al. investigated community‐dwelling older adults with depression. Through primarily psychological interventions, they found that remission of depressive symptoms was associated with ADL recovery. However, partial improvement in depressive symptoms was not linked to ADL recovery,^47^ which differs from our findings. Another study^50^, ^51^ demonstrated improvements in both depression and ADL after interdisciplinary intervention, including a daily 20‐min physical therapy session (mean hospital stay: 10.1 days), in patients with fractures. However, this study did not limit its target population to patients with depression, nor did it examine the relationship between the degree of depressive symptom improvement and ADL recovery. Depressive symptoms, such as apathy and loss of motivation,^52^, ^53^, ^54^, ^55^ can hinder rehabilitation outcomes.^56^ In this study, improvement in depressive symptoms likely increased patients’ motivation for rehabilitation, enhancing its effectiveness. Even patients in the lowest rehabilitation intensity group received daily rehabilitation sessions of 122.1 min (median hospital stay: 67.0 days). This intensive and sustained rehabilitation may have amplified the benefits of partial improvement in depressive symptoms, leading to significant cumulative effects. In addition, behavioral and biological mechanisms have been proposed to explain the impact of depression on physical disability. Behaviorally, depression can impair adherence to medical care and self‐management, exacerbating disability. Biologically, depression may disrupt the hypothalamic–pituitary–adrenal axis, sympathetic nervous system, and immune function, leading to physical deterioration.^57^ In convalescent rehabilitation wards, patients frequently interact with healthcare professionals. Therapists provide face‐to‐face rehabilitation sessions, medical doctors conduct regular rounds, and nurses deliver daily care. These interactions ensure high levels of engagement with medical staff, which likely improves adherence to medical and self‐management instructions. Improved adherence may prevent further functional decline, even with partial improvement in depression. This may enable patients to fully benefit from rehabilitation, allowing ADL recovery to progress effectively.

Pharmacotherapy is a common primary treatment for depressive disorders.^58^, ^59^ However, antidepressants are associated with adverse events, such as sedation, nocturia, orthostatic hypotension, and postural reflex impairment, which increase the risk of falls.^60^, ^61^, ^62^, ^63^, ^64^ In addition, several antidepressants have been reported to have a high frequency of somnolence^65^ and daytime sleepiness^61^ in clinical trials. Daytime sleepiness is a predictor of lower rates of physical function recovery in the rehabilitation of older patients, and is associated with lower improvement in ADL.^66^ In this study, antidepressant users may have experienced lower rates of physical function recovery in rehabilitation due to daytime sleepiness and somnolence, which may have resulted in low improvement in ADL.

This study provides new insights into the relationship between rehabilitation and improvement in depressive symptoms in older patients with fractures. Under high‐frequency rehabilitation, partial improvement in depressive symptoms may promote rehabilitation effects and directly contribute to ADL recovery. However, a bidirectional relationship between improvement in depressive symptoms and ADL recovery has been reported.^67^ As this study was retrospective and observational, establishing causality between these factors is challenging. Nevertheless, it highlights the important finding that rehabilitation can play a significant role in managing depression in older patients with fractures, even in medical facilities without psychiatric specialists. While pharmacotherapy remains a common and effective treatment for depressive symptoms, its associated adverse effects underscore the importance of avoiding over‐reliance on this approach. The findings of this study show that rehabilitation is effective in the management of depression, and an approach based on this relationship may contribute to solving problems related to depression in older patients with fractures. Additionally, expanding treatment options for depressive symptoms, including tailored rehabilitation strategies, would enable customized care for individual patients, potentially enhancing treatment efficacy. Such efforts may ultimately lead to improved long‐term prognosis for older patients with fractures.

This study has some limitations. First, as a retrospective observational study, it cannot establish causality between the improvement in depressive symptoms and ADL recovery. Prospective studies are needed to demonstrate this relationship. Second, as this study was conducted at a single institution, its findings may not be generalizable. Validation through multicenter studies is necessary to confirm these results across different healthcare settings.

## CONCLUSION

This study showed that both remission, and partial improvement, in depressive symptoms was associated with ADL recovery in older patients with fractures and depression. These results suggest that comprehensive interventions, including the management of depression, in addition to that of physical function, may enhance rehabilitation effectiveness. This may improve ADL and long‐term prognosis in older patients with fractures.

## AUTHOR CONTRIBUTIONS

Shunji Araki, Takahiro Ogawa, and Yoshikazu Takaesu contributed to the conception and design of this study. Shunji Araki collected the data, conducted the statistical analysis, and drafted the manuscript. Takahiro Ogawa and Yoshikazu Takaesu interpreted the data and critically reviewed the manuscript. Yoshikazu Takaesu supervised the overall study process. All authors have read and approved the final manuscript.

## CONFLICT OF INTEREST STATEMENT

The authors declare no conflicts of interest.

## ETHICS APPROVAL STATEMENT

This study was approved by the Ethics Review Committee for Life Sciences and Medical Research Involving Human Subjects, University of the Ryukyus (approval ID: 23‐2256‐00‐00‐00). This study was retrospective in design, making it difficult to obtain individual informed consent; a waiver of informed consent was granted by the Ethics Review Committee. However, an opt‐out procedure was used to provide all participants with the opportunity to exclude their data from the study analysis.

## PATIENT CONSENT STATEMENT

This study was retrospective in design, making it difficult to obtain individual informed consent; a waiver of informed consent was granted by the Ethics Review Committee. However, an opt‐out procedure was used to provide all participants with the opportunity to exclude their data from the study analysis.

## CLINICAL TRIAL REGISTRATION

N/A.

## Data Availability

The data supporting this study are not publicly available due to ethical and privacy considerations. However, access to the data can be granted on a reasonable request basis, subject to ethical approval. For further inquiries, please contact the corresponding author.
